# Comparative associations of MASLD and MAFLD with the presence and severity of coronary artery calcification

**DOI:** 10.21203/rs.3.rs-3979461/v1

**Published:** 2024-03-05

**Authors:** Min Kyu Kang, Jeong Song, Rohit Loomba, Soo Park, Won Tak, Young Kweon, Yu Lee, Jung Gil Park

**Affiliations:** Yeungnam University; Catholic University of Daegu; University of California, San Diego; Kyungpook National University; Kyungpook National University; Kyungpook National University; Kyungpook National University; Yeungnam University

**Keywords:** metabolic dysfunction-associated steatotic liver disease, metabolic dysfunction-associated fatty liver disease, coronary artery calcification, cardiovascular disease

## Abstract

We aimed to compare the associations of metabolic dysfunction-associated steatotic liver disease (MASLD) and metabolic dysfunction-associated fatty liver disease (MAFLD) with coronary artery calcification (CAC). Patients who simultaneously underwent ultrasonography to diagnose hepatic steatosis and cardiac computed tomography to detect CAC were included. The presence and severity of CAC were defined with CAC-score thresholds of >0 and > 300, respectively, and patients were divided into the following groups: no MASLD or MAFLD (reference), MASLD-only, MAFLD-only, and overlapping groups. Overall, 1,060/2,773 (38.2%) patients had CAC, of which 196 (18.5%) had severe CAC. The MASLD and MAFLD prevalence rates were 32.6% and 45.2%, respectively, with an overlap of 30.7%. In an ASCVD risk score-adjusted model, both MASLD (adjusted odd ratios [aOR], 1.21; 95% confidence interval [CI], 1.02–1.44; p = 0.033) and MAFLD (aOR, 1.20; 95% CI, 1.01–1.42, *p* = 0.034) were associated with CAC, whereas only MASLD (aOR, 1.38; 95% CI, 1.01–1.89, *p* = 0.041) was associated with severe CAC. Compared to the reference group, the overlapping group showed an association with CAC (aOR, 1.22; 95% CI, 1.01–1.47; p = 0.038); however, the MASLD and MAFLD subgroups did not differ in their association with CAC. MASLD may predict a higher risk of ASCVD more effectively than MAFLD.

## Introduction

Non-alcoholic fatty liver disease (NAFLD) is defined as hepatic steatosis of ≥ 5% without the evidence of excessive alcohol consumption and other chronic liver diseases^1,2^. It has a global prevalence of approximately 30–35% and coexists with obesity, insulin resistance, and diabetes, which accounts for a large socioeconomic burden^1,3^. The prognosis of NAFLD varies depending on the degree of fibrosis, with cardiovascular outcomes being the primary cause of mortality^1,4^. However, due to the heterogenous pathogenesis of NAFLD and uncertainty regarding its classification, NAFLD nomenclature underestimates the significance of metabolic comorbidities and extrahepatic manifestations^5^.

The nomenclature for metabolic dysfunction-associated fatty liver disease (MAFLD) was introduced in 2019^6^. Compared with NAFLD, MAFLD is a positive diagnosis that includes other chronic liver diseases and is categorised into three stratified subtypes based on diabetes, obesity, and other metabolic components. Several studies have indicated that the MAFLD criteria may identify a higher risk of cardiovascular disease (CVD) than NAFLD^7–9^. However, different subtypes of MAFLD—such as diabetic and non-diabetic MAFLD—have different CVD morbidity and mortality rates^10,11^. Further drawbacks of the MAFLD nomenclature include heterogeneous pathogeneses resulting from mixed aetiologies and a restricted comprehension of the disease’s natural history due to more lenient alcohol consumption^12,13^.

In 2023, metabolic dysfunction-associated steatotic liver disease (MASLD) emerged as the new nomenclature for steatotic liver disease (SLD), replacing both types of fatty liver disease, which have potentially stigmatising descriptions^12,13^. MASLD is defined as the presence of at least one of five cardiometabolic criteria of hepatic steatosis without any other apparent causes^12,13^. This umbrella term provides a comprehensive definition of SLD, with a focus on metabolic dysfunction^12,13^. Due to the recent change in the nomenclature, the relationship between MASLD and the risk of CVD remains uncertain. Given the high predictive value of MAFLD for predicting the risk of CVD, it is important to elucidate the association between MASLD and CVD risk and compare it to that of MAFLD.

Quantitatively scoring coronary artery calcification (CAC) is a reliable, non-invasive tool for detecting the presence of coronary artery disease and assessing the severity of CVD in asymptomatic individuals. This score is closely correlated with the degree of atherosclerosis and cardiovascular mortality, regardless of traditional risk factors^14,15^.

This study aimed to investigate the potential of MASLD and MAFLD as predictors of CVD risk based on the presence and severity of CAC. Additionally, we compared the associations between the MASLD and MAFLD subgroups with respect to the risk of CVD.

## Results

### Baseline characteristics

Table 1 summarises the baseline characteristics of the patients with CAC (CAC group) and those without CAC (no-CAC group). Of the 2,773 participants, 1,060 (38.2%) were categorised into the CAC group. Notably, compared to patients without CAC, those with CAC were older (55.0 [49.0–61.0] vs. 60.0 [55.0–66.0] years, *p* < 0.001), were more likely to be male (61.5% vs. 79.6%, *p* < 0.001), had a higher body mass index (BMI) (23.7 ± 2.9 vs. 24.0 ± 2.8 kg/m^2^, *p* = 0.001), were more likely to have diabetes (13.0% vs. 27.5%, *p* = 0.002), were more likely to be current smokers (50.1% vs. 67.3%, *p* < 0.001), had lower platelet counts (243.5 ± 59.4 vs. 233.2 ± 54.1, *p* < 0.001), had a higher prevalence of statin usage (14.1% vs. 27.9%, *p* < 0.001), had high proportions of advanced fibrosis (3.2% vs. 7.3%, *p* < 0.001), and had higher atherosclerotic cardiovascular disease (ASCVD) risk scores (5.6 [2.4–10.8] vs. 13.1 [7.4–20.1], *p* < 0.001). In the CAC group, the median CAC score was 63.9 and the prevalence rates of mild, moderate, and severe CAC were 60.1 %, 21.4%, and 18.5%, respectively.

**Figure 1:**
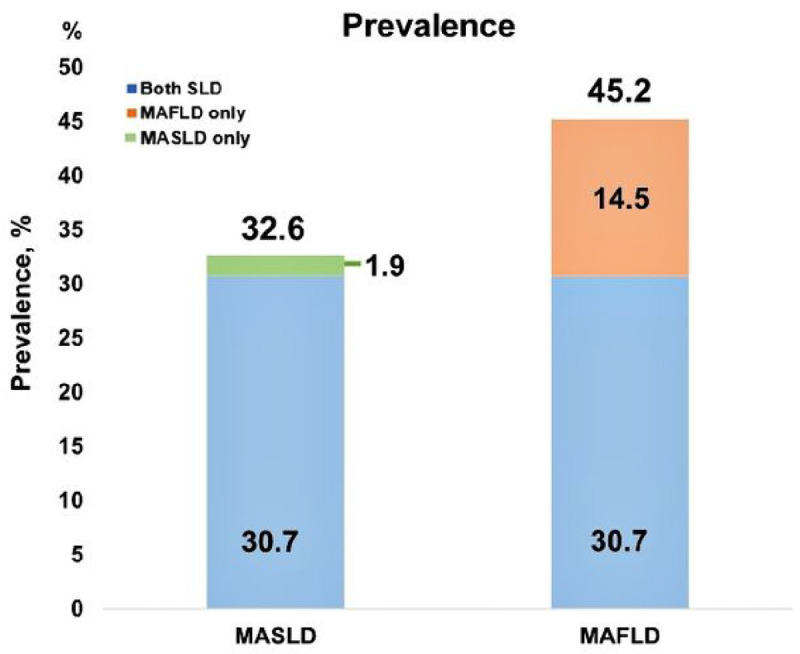
The prevalence of MASLD and MAFLD.

In contrast, the prevalence rates of MASLD and MAFLD were 32.6% and 45.2%, respectively. Furthermore, the prevalence of satisfying both MASLD and MAFLD criteria was 30.7%, compared with 1.9% for MASLD alone and 14.5% for MAFLD alone (Fig. 1).

### Adjusted associations of CAC based on the presence or absence of MASLD or MAFLD

Table 2 shows the adjusted risk of CAC according to the presence or absence of MASLD or MAFLD. Compared with the no-MASLD group, the patients with MASLD remained at higher risk of CAC after adjustment for age and sex (Model 1: adjusted odds ratio (aOR), 1.36; 95% confidence interval (CI), 1.13–1.63; *p* = 0.001); smoking status, use of statins, diabetes, and advanced fibrosis (Model 2: aOR, 1.25; 95% CI, 1.04–1.51; *p* = 0.018); and ASCVD risk score (Model 3: aOR, 1.21; 95% CI, 1.02–1.44; *p* = 0.033).

Similarly, compared with the no-MAFLD group, the patients with MAFLD had a higher risk of CAC after adjustment for age and sex (Model 1: aOR, 1.48; 95% CI, 1.25–1.75; *p* < 0.001); smoking status, use of statins, diabetes, and advanced fibrosis (Model 2: aOR, 1.33; 95% CI, 1.12–1.58; *p* = 0.001); and ASCVD risk score (Model 3: aOR, 1.20; 95% CI, 1.01–1.42; *p* = 0.034).

### Adjusted associations of severe CAC based on the presence or absence of MASLD or Metabolic dysfunction-associated fatty liver disease

Table 3 shows the adjusted associations of CAC severity in patients with MASLD and MAFLD compared to those without. Compared with the no-MASLD group, the patients with MASLD maintained a higher risk of severe CAC after adjustment for age and sex (Model 1: aOR, 1.60; 95% CI, 1.16–2.22; *p* = 0.005); smoking status, use of statins, diabetes, and advanced fibrosis (Model 2: aOR, 1.49; 95% CI, 1.07–2.08; *p* = 0.017); and ASCVD risk score (Model 3: aOR, 1.38; 95% CI, 1.01–1.89; *p* = 0.041).

Unlike the no-MAFLD group, the patients with MAFLD had an increased risk of severe CAC only after adjustment for age and sex (Model 1: aOR, 1.49; 95% CI, 1.10–2.03; *p* = 0.011), whereas no association with severe CAC was observed upon further adjustment (Table 3).

### Adjusted associations of CAC according to subgroups of different steatotic liver statuses

We assessed the effect of CAC on four subgroups of patients categorised according to their steatotic liver status (Table 4). Compared with the group with neither MASLD nor MAFLD, both the MASLD and MAFLD groups were associated with an increased risk of CAC after adjustment for age and sex (Model 1: aOR, 1.48; 95% CI, 1.22–1.81; *p* < 0.001); smoking status, use of statins, diabetes, and advanced fibrosis (Model 2: aOR, 1.34; 95% CI, 1.09–1.64; *p* = 0.005); and ASCVD risk score (Model 3: aOR, 1.22; 95% CI, 1.01–1.47; *p* = 0.038), whereas the MASLD-only and MAFLD-only groups were not.

**Figure 2:**
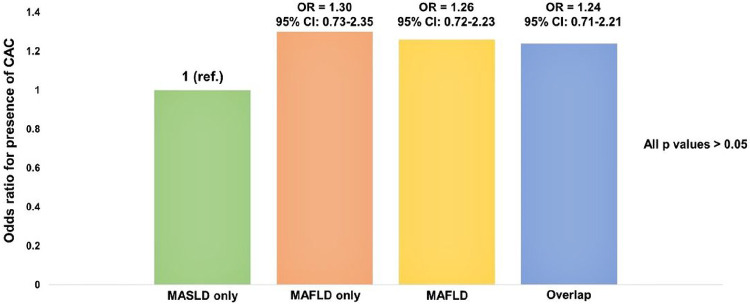
Odds ratios for the presence of CAC between the subgroups of MASLD and MAFLD.

To directly compare the impact of CAC on each subgroup of patients with MASLD and/or MAFLD, we established the MASLD-only group as a reference (Fig. 2). Compared with the MASLD-only group, the MAFLD-only, MAFLD, and both MASLD and MAFLD groups were not significantly associated with CAC (all *p* > 0.05).

## Discussion

In this study, we identified an association between MASLD and the presence and severity of CAC. In addition, we found that MASLD has a cardiovascular risk comparable to that of MAFLD but tends to outperform MAFLD in the assessment of CVD severity using the CAC score. This suggests that MASLD, as a novel nomenclature, has the potential to replace MAFLD when predicting the CVD risk.

This study has some clinical implications. First, the prevalence of MASLD in the two health-promotion centre populations was 32.6%. In two recent studies, the prevalence of MASLD was 33.4% in a Brazilian cohort and 14.87% in a Chinese cohort^16,17^. These discrepancies are possibly due to differences in the enrolled populations, including the timing of cohort recruitment and ethnic differences. Moreover, compared to a previous meta-analysis that reported the prevalence of MAFLD to be 38.77% (95% CI, 32.94–44.95%)^18^, the prevalence of MAFLD in our study was slightly higher at 45.2%. Additionally, the prevalence of overlap between MASLD and MAFLD in our study was 30.7%, which was comparable to that between traditional NAFLD and MAFLD (27.4%) in a previous study^7^. Further research is required to investigate the prevalence of MASLD in a large population cohort that reflects sex- and ethnicity-specific characteristics.

When compared with the non-MASLD or non-MAFLD groups, both the MASLD (odds ratio [OR], 1.21; 95% CI, 1.02–1.44) and MAFLD (OR, 1.20; 95% CI, 1.01–1.42) groups showed an ASCVD risk score-adjusted association with the presence of CAC. Similarly, both MAFLD and NAFLD have been associated with an approximately 1.2 to 2–fold increased risk of CVD in previous studies^9,19,20^. A previous meta-analysis revealed an association between the presence of CAC (OR, 1.272; 95% CI, 1.114–1.452), severe CAC defined as CAC >100 (OR, 1.242; 95% CI, 1.017–1.516) and NAFLD, irrespective of traditional risk factors, which is similar to our results^21^.

However, in the present study, MASLD showed an ASCVD risk score-adjusted association with severe CAC (OR, 1.38; 95% CI, 1.01–1.89), whereas MAFLD did not (OR, 1.15; 95% CI, 0.84–1.57). These results may be related to the heterogeneous subtypes of MAFLD. Among the three MAFLD subtypes, diabetes is the most well-established risk factor for CAC, and the MAFLD-diabetic subtype is associated with an increased risk of all-cause mortality and CVD^11,22^. However, recent research indicates that the MAFLD-overweight/obese subtype does not increase the risk of all-cause mortality or cardiovascular morbidity^10,11^. Additionally, the MAFLD-lean subtype with less than two metabolic factors is not associated with CAC^11^. To investigate the association between the presence of CAC and MAFLD subtypes, we divided the MAFLD group into diabetic and non-diabetic subgroups. We found that the presence of CAC was significantly associated with the MAFLD-diabetic subtype when adjusted for age and sex, but not with the MAFLD-non-diabetic subtype when unadjusted (Supplementary Table 1). This implies that the associations of overall MAFLD with severe CAC may differ due to its heterogeneous subtypes, indicating that MASLD may be more effective than MAFLD when evaluating the severity of CVD. Moreover, a previous study suggested that light and moderate alcohol consumption may have a protective effect on CVD mortality^23^. Therefore, MAFLD may be associated with a reduced risk of CAC due to the higher proportion of light-to-moderate drinkers than in patients with MASLD; however, further research is required to reflect the exact impact of alcohol intake on CAC.

Furthermore, our subgroup analysis revealed that only overlap between the MASLD and MAFLD groups was associated with the presence of CAC when neither group was used as a reference. However, the MASLD-only and MAFLD-only groups showed no association with CAC, which is a distinctive finding. Additionally, there was no evidence to suggest the superiority of CAC in the definition of either type of SLD through comparisons between the MASLD-only and MAFLD subgroups. Contrary to our findings, previous studies comparing the efficacies of NAFLD and MAFLD in predicting the risk of CVD showed that the MAFLD group and the overlap between the NAFLD and MAFLD groups were associated with an increased risk of CVD^7,9^. In one study, the aOR for a high ASCVD risk in the MAFLD-only and MAFLD groups were 3.26 and 3.14, respectively, using the NAFLD-only group as a reference group^24^. In addition, previous studies have shown that the MAFLD definition is more effective than the NAFLD definition when predicting the prognosis of other diseases^25,26^.

The subgroups in this study were characterised as follows: the MAFLD-only group included patients with metabolic risk factors and other aetiologies such as alcohol consumption and chronic hepatitis C, whereas the MASLD-only group had a comparatively favourable metabolic status, with all metabolic risk factors scoring 1. Compared to the definition of NAFLD, the MASLD definition is believed to more directly and comprehensively include the metabolic components of MAFLD, thereby homogenising the overlap of the metabolically unfavourable portions. In other words, groups other than the homogeneous groups (NAFLD-only and MAFLD-only) showed a favourable metabolic status when compared with the overlap group. Future studies should use large longitudinal data to determine the comparative associations between MASLD, MAFLD, and CVD risk.

In this study, we also compared the CAC risk of each MASLD and MAFLD adjusted for the presence of advanced fibrosis, defined as Fib-4 ≥ 2.67, in addition to traditional risk factors. NAFLD is known to be associated with both fatal and nonfatal CVD events, depending on the stage of liver fibrosis^27^. We have previously shown that significant fibrosis, as defined by MRE, is associated with the presence of CAC in patients with NAFLD^28^. Furthermore, considering that MAFLD may better identify significant liver fibrosis than NAFLD, the assessment of the fibrosis stage may be an important consideration when comparing CVD risk between MAFLD and MASLD^29^.

Our study had several limitations. First, this was a cross-sectional retrospective study that does not establish causality with CVD in patients with SLD. Second, we only included individuals who voluntarily visited the health-promotion centre and could afford the full test, including cardiac CT, which may have resulted in a selection bias. Third, this study was conducted in South Korea, potentially restricting the generalisability of the findings to the entire population. Therefore, prospective, well-designed longitudinal studies are warranted to validate the causal relationship between MASLD and the incident and prevalent CVD risks. Fourth, the subjects enrolled in this study did not meet the criteria for MAFLD owing to the lack of homeostasis model assessment of insulin resistance (HOMA-IR) measurements. However, owing to the high cost and complexity of this procedure, the use of IR markers as the primary test for identifying MAFLD in the general population is challenging. In contrast, the MASLD can identify and assess risk factors through simple blood tests and demographic measurements.

In conclusion, beyond being associated with the presence of CAC, independent of traditional risk factors, MASLD was more effective than MAFLD in identifying severe CAC. Moreover, compared with the definition of MAFLD, the new MASLD nomenclature might not show any gap in the prediction of CVD risk using the CAC score. Therefore, the assessment and stratification of CAC may be useful in predicting a high risk of CVD events in patients with MASLD.

## Methods

### Patients

This cross-sectional, multicentre, retrospective study enrolled individuals who underwent comprehensive health screening using abdominal ultrasonography and cardiac computed tomography (CT) between January 2017 and December 2021 at two health-promotion centres in Daegu, South Korea. In total, 2,906 individuals who underwent abdominal ultrasonography and cardiac CT were examined. The exclusion criteria were a documented history of significant CVD or cardiac intervention and missing or inadequate data. Of the 2,906 patients, none experienced CVD events; however, 133 individuals had missing or inadequate data. Therefore, a total of 2,773 participants were enrolled in this study.

### Definition and classification of MASLD and MAFLD

Hepatic steatosis was assessed based on the following criteria: increased echogenicity of the liver compared with that of the renal cortex, deep attenuated beam, and blurred intrahepatic vessels on abdominal ultrasound^30^. MASLD is defined as hepatic steatosis in addition to at least one of the five cardiometabolic criteria^12,13^, whereas MAFLD is defined as hepatic steatosis in addition to the presence of one of the following metabolic conditions: diabetes, overweight/obesity, or evidence of at least two out of seven metabolic abnormalities^6^. Due to all the enrolled patients being Korean, waist circumference and BMI were determined using cut-off values for Asians. HOMA-IR values was excluded due to the absence of fasting insulin levels. Individuals who did not fulfil the MASLD or MAFLD criteria were classified into the no-MASLD or no-MAFLD groups, respectively. Moreover, individuals who met neither the MASLD nor the MAFLD criteria were classified into the neither MASLD nor MAFLD group.

**Figure 3:**
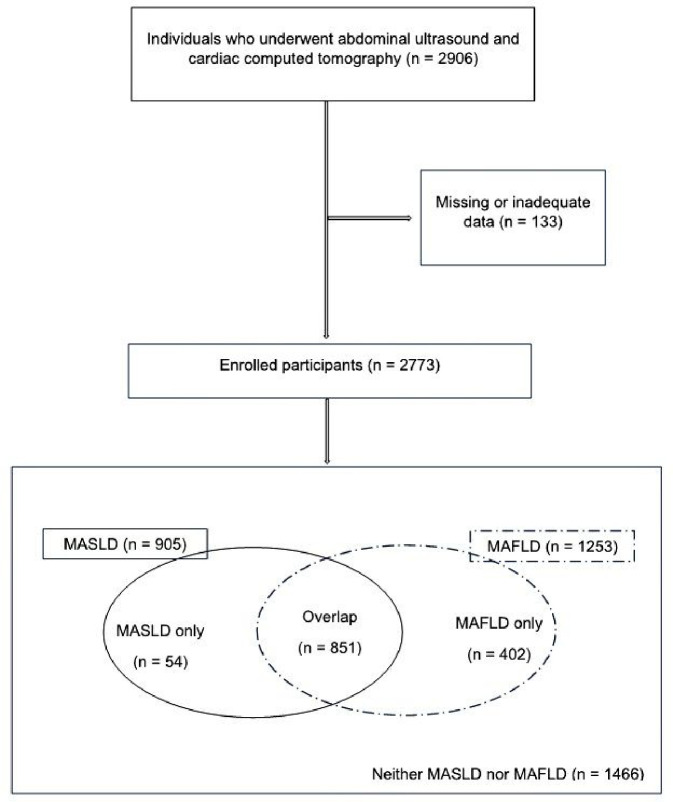
Flow charts and Venn diagram of the enrolled participants.

Of the 2,773 participants, 905 met the criteria for MASLD, 1,253 met the criteria for MAFLD, 1,466 had neither MASLD nor MAFLD, and 851 met the criteria for overlap between MAFLD and MASLD. Moreover, 54 patients did not fulfil the MAFLD criteria and were diagnosed with MASLD only, while 402 did not meet the MASLD criteria and were diagnosed with MAFLD only (Fig. 3).

### Quantification of CAC using cardiac CT

Two non-contrast cardiac prospective electrocardiogram-gated volumetric CT scans were performed at each institution using a 256-slice CT scanner (Revolution; GE Healthcare) and a 320-slice CT scanner (SOMATOM Force, Siemens Healthineers). At the end of inspiration, individuals were instructed to hold their breath while undergoing a scan ranging from the base of the heart to the carina. The tube voltage ranged from 100 to 120 kVp. Five filter revolutions were used for reconstruction, resulting in 2.5 mm-thick reconstruction slices. Subsequently, the CAC was calculated for each cardiac CT protocol. The CAC scores were calculated using the Agatston scoring method through independent postprocessing software (TeraRecon and Syngo.via; Siemens Healthineers). The presence of CAC was determined by a CAC score of > 0, whereas severe CAC was defined as a score of >300^14,31^.

### Statistical analysis

Data are expressed as means with standard deviations, medians with interquartile ranges, or numbers and percentages, as appropriate. Continuous data were analysed using the Student *t*-test or Mann-Whitney U test, and categorical data were compared using the chi-square test or Fisher exact test after testing for normality. To evaluate the associations of MASLD and MAFLD with the presence or severity of CAC, adjusted logistic regression models with stepwise backward elimination of OR were assessed. Model 1 was adjusted for age and sex, whereas Model 2 was adjusted for smoking, statin use, diabetes, and advanced fibrosis, defined as a fibrosis-4 index > 2.67 after adjustment for Model 1. To avoid multicollinearity, Model 3 was adjusted for the ASCVD risk score only, which included age, sex, race, smoking status, blood pressure, use of antihypertensive medication, diabetes status, and levels of total and high-density lipoprotein cholesterol.

All statistical analyses were performed using the R software (version 4.1.0; R Core Team, 2021; R Foundation for Statistical Computing, Vienna, Austria), and statistical significance was set at *p* < 0.05.

## Figures and Tables

**Table 1:** Baseline characteristics of the patients.

	Absence of CAC n = 1,713 (61.8%)	Presence of CAC n = 1,060 (38.2%)	*p*-value
Demographic profile			
Age (yr)	55.0 [49.0–61.0]	60.0 [55.0–66.0]	< 0.001
Male	1054 (61.5)	844 (79.6)	< 0.001
BMI (kg/m^2^)	23.7 ± 2.9	24.0 ± 2.8	0.001
Waist circumference (cm)	84.8 ± 9.1	87.1 ± 8.5	< 0.001
Diabetes	223 (13.0)	291 (27.5)	0.002
Current smoker	858 (50.1)	713 (67.3)	< 0.001
Liver function tests			
AST (U/L)	27.6 ± 15.5	29.4 ± 16.2	0.003
ALT (U/L)	27.8 ± 19.5	29.3 ± 19.9	0.055
Platelet counts (×10^9^/L)	243.5 ± 59.4	233.2 ± 54.1	< 0.001
Albumin (g/dL)	4.5 ± 0.4	4.7 ± 0.6	0.197
Total bilirubin (mg/dL)	0.8 ± 1.7	0.8 ± 0.4	0.539
Metabolic profile (mg/dL)			
Fasting glucose	98.5 ± 22.2	105.4 ± 27.8	< 0.001
Total cholesterol	191.9 ± 38.3	182.5 ± 40.5	< 0.001
TG	124.4 ± 89.6	130.1 ± 83.3	0.091
HDL-C	56.5 ± 15.4	53.6 ± 14.4	< 0.001
LDL-C	128.8 ± 46.0	120.5 ± 38.8	< 0.001
Use of statin	241 (14.1)	296 (27.9)	< 0.001
Fib-4 ≥ 2.67	54 (3.2)	77 (7.3)	< 0.001
Cardiovascular profile			
ASCVD risk score	5.6 [2.4–10.8]	13.1 [7.4–20.1]	< 0.001
CAC score		63.9 [14.8–202.5]	
CAC grade			
Mild (CAC score, 1–99)		637 (60.1)	
Moderate (CAC score, 100–299)		227 (21.4)	
Severe (CAC score, ≥ 300)		196 (18.5)	

Data are presented as mean ± standard deviation, median [interquartile range], or number (%). Presence of CAC is defined as a CAC score > 0. CAC, coronary artery calcification; BMI, body mass index; AST, aspartate aminotransferase; ALT, alanine aminotransferase; TG, triglycerides; HDL-C, high-density lipoprotein cholesterol; LDL, low-density lipoprotein cholesterol; Fib-4, fibrosis-4 index; ASCVD, atherosclerotic cardiovascular disease.

**Table 2:** Adjusted risk for CAC according to the presence or absence of MASLD or MAFLD.

	Number	Presence of CAC	Model 1 aOR (95% CI)	Model 2 aOR (95% CI)	Model 3^†^ aOR (95% CI)
MASLD					
No-MASLD	1868	664	1 (ref.)	1 (ref.)	1 (ref.)
MASLD	905	396	1.36 (1.13–1.63)*	1.25 (1.04–1.51)*	1.21 (1.02–1.44)*
MAFLD					
No-MAFLD	1520	503	1 (ref.)	1 (ref.)	1 (ref.)
MAFLD	1253	557	1.48 (1.25–1.75)*	1.33 (1.12–1.58)*	1.20 (1.01–1.42)*

* A *p*-value < 0.05.

Model 1, adjusted for age and sex; Model 2, Model 1 + smoking, use of statins, diabetes, and advanced fibrosis; Model 3: ^†^ASCVD risk score. Presence of CAC is defined as a CAC score > 0 and advanced fibrosis is defined as a fibrosis-4 index ≥ 2.67.

† The ASCVD risk score includes age, sex, race, smoking status, blood pressure, medication use for hypertension, diabetes status, total cholesterol, and high-density lipoprotein cholesterol.

CAC, coronary artery calcification; MASLD, metabolic dysfunction-associated steatotic liver disease; MAFLD, metabolic dysfunction-associated fatty liver disease; aOR, adjusted odds ratio; CI, confidence interval; ref., reference; ASCVD, atherosclerotic cardiovascular disease.

**Table 3:** Adjusted risk of severe CAC according to the presence or absence of MASLD or MAFLD.

	Number	Severe CAC	Model 1 aOR (95% CI)	Model 2 aOR (95% CI)	Model 3^†^ aOR (95% CI)
MASLD					
No-MASLD	1868	115	1 (ref.)	1 (ref.)	1 (ref.)
MASLD	905	81	1.60 (1.16–2.22)*	1.49 (1.07–2.08)*	1.38 (1.01–1.89)*
MAFLD					
No-MAFLD	1520	86	1 (ref.)	1 (ref.)	1 (ref.)
MAFLD	1253	110	1.49 (1.10–2.03)*	1.33 (0.97–1.82)	1.15 (0.84–1.57)

* A *p*-value < 0.05.

Model 1, adjusted for age and sex; Model 2, Model 1 + smoking, use of statins, diabetes, and advanced fibrosis; Model 3: ^†^ASCVD risk score. Presence of severe CAC is defined as a CAC score > 300 and advanced fibrosis is defined as a fibrosis-4 index ≥ 2.67.

† The ASCVD risk score includes age, sex, race, smoking status, blood pressure, medication use for hypertension, diabetes status, total cholesterol, and high-density lipoprotein cholesterol.

CAC, coronary artery calcification; MASLD, metabolic dysfunction-associated steatotic liver disease; MAFLD, metabolic dysfunction-associated fatty liver disease; aOR, adjusted odds ratio; CI, confidence interval; ref., reference; ASCVD, atherosclerotic cardiovascular disease.

**Table 4:** Adjusted risk of CAC according to the subgroups of different steatotic liver statuses.

	Number	Presence of CAC	Model 1 aOR (95% CI)	Model 2 aOR (95% CI)	Model 3^†^ aOR (95% CI)
Neither MASLD nor MAFLD	1,466	482	1 (ref.)	1 (ref.)	1 (ref.)
MASLD only	54	21	1.30 (0.73–2.25)	1.30 (0.73–2.25)	1.30 (0.73–2.25)
MAFLD only	402	182	1.45 (1.13–1.86)*	1.29 (0.99–1.67)	1.11 (0.86–1.43)
Both MASLD and MAFLD	851	375	1.48 (1.22–1.81)*	1.34 (1.09–1.64)*	1.22 (1.01–1.47)*

* A *p*-value < 0.05.

Model 1, adjusted for age and sex; Model 2, Model 1 + smoking, use of statins, diabetes, and advanced fibrosis; Model 3: ^†^ASCVD risk score. Presence of CAC is defined as a CAC score > 0 and advanced fibrosis is defined as a fibrosis-4 index ≥ 2.67.

† The ASCVD risk score includes age, sex, race, smoking status, blood pressure, medication use for hypertension, diabetes status, total cholesterol, and high-density lipoprotein cholesterol.

CAC, coronary artery calcification; aOR, adjusted odds ratio; CI, confidence interval; MASLD, metabolic dysfunction-associated steatotic liver disease; MAFLD, metabolic dysfunction-associated fatty liver disease; ref., reference.

## Data Availability

The data supporting findings of this study are available from the corresponding authors upon reasonable request.

## Abbreviations

ALT: alanine aminotransferase
aOR: adjusted odds ratio
ASCVD: atherosclerotic cardiovascular disease
AST: aspartate aminotransferase
BMI: body mass index
CAC: coronary artery calcification
CI: confidence interval
CT: computed tomography
CVD: cardiovascular disease
Fib-4: fibrosis-4 index
HDL-C: high-density lipoprotein cholesterol
HOMA-IR: Homeostasis model assessment of insulin resistance
LDL: low-density lipoprotein cholesterol
MAFLD: metabolic dysfunction-associated fatty liver disease
MASLD: metabolic dysfunction-associated steatotic liver disease
SLD: steatotic liver disease
TG: Triglycerides
